# Down‐regulation of OIP5‐AS1 inhibits obesity‐induced myocardial pyroptosis and miR‐22/NLRP3 inflammasome axis

**DOI:** 10.1002/iid3.1066

**Published:** 2023-10-27

**Authors:** Qingxiong Yue, Yan Liu, Jun Ji, Tao Hu, Tong Lin, Shuang Yu, Shijun Li, Nan Wu

**Affiliations:** ^1^ Department of Ultrasound Dalian Municipal Central Hospital Dalian Liaoning Province China; ^2^ Department of Ultrasound Dalian Women and Children's Medical Group Dalian Liaoning Province China; ^3^ Department of Central Laboratory Dalian Municipal Central Hospital Dalian Liaoning Province China; ^4^ Department of Central Laboratory First Affiliated Hospital of China Medical University Shenyang Liaoning Province China; ^5^ Department of Cardiology Dalian Municipal Central Hospital Dalian Liaoning Province China

**Keywords:** inflammation, myocardial injury, obesity, opa‐interacting protein 5‐antisense transcript 1, pyroptosis

## Abstract

**Background:**

Obesity can induce myocardial pyroptosis, but the exact mechanism is still unknown. A recent study reported the association of opa‐interacting protein 5‐antisense transcript 1 (OIP5‐AS1), an evolutionarily conserved long noncoding RNA, with pyroptosis. Therefore, this study aimed to investigate the role of OIP5‐AS1 in obesity‐induced myocardial pyroptosis.

**Methods:**

OIP5‐AS1 was downregulated in H9c2 cells, followed by treatment with 400 μM palmitic acid (PA). Propidium iodide (PI) staining, lactic dehydrogenase (LDH) release assay, caspase‐1 activity assay, IL‐1β, and IL‐18 activity assay were performed to detect pyroptotic phenotype. The interaction between OIP5‐AS1 and microRNAs (miRNAs) was analyzed using RNA pull‐down and luciferase assay. The effect of OIP5‐AS1 knockdown in high‐fat diet (HFD)‐induced obesity rat on cardiac function, myocardial hypertrophy, fibrosis, and remodeling was evaluated.

**Results:**

Fat deposition was observed in cardiomyocytes 24 h after PA treatment; moreover, PA‐treated cardiomyocytes showed significant increase in the rate of pyroptotic cells, release of LDH, protein expressions of NLRP3 and cleaved caspase‐1, and the activity of caspase‐1, IL‐1β, and IL‐18 as well as OIP5‐AS1 expression. These findings suggested that PA activated pyroptosis and induced OIP5‐AS1 expression in cardiomyocytes. Moreover, OIP5‐AS1 knockdown inhibited PA‐induced pyroptosis. Mechanistically, OIP5‐AS1 was found to specifically bind to miR‐22 and to regulate NLRP3 inflammasome‐mediated pyroptosis via miR‐22. Furthermore, OIP5‐AS1 knockdown ameliorated HFD‐induced cardiac dysfunction, myocardial hypertrophy, fibrosis, remodeling, and pyroptosis.

**Conclusion:**

Our results revealed that downregulation of OIP5‐AS1 can inhibit obesity‐induced myocardial pyroptosis via miR‐22/NLRP3 inflammasome axis. This finding lays a foundation of gene therapy for heart disease targeting OIP5‐AS1.

## Graphical abstract

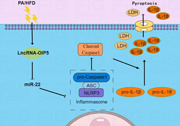

## 1 INTRODUCTION

Obesity is a chronic, mild inflammatory state associated with increased levels of inflammatory biomarkers, such as tumor necrosis factor‐α (TNF‐α), interleukin 6 (IL‐6), and C‐reactive protein.^1^, ^2^ A growing body of evidence has demonstrated that obesity can induce myocardial chronic inflammation due to the accumulation of a large number of lipids and their oxidation intermediates in cardiomyocytes,^3^ which further causes myocardial dysfunction, myocardial hypertrophy, fibrosis and remodeling, and ultimately leads to heart failure.^4^, ^5^ In recent studies, inhibition of inflammation by genetic or pharmacological approach was found to prevent obesity‐induced heart injury in vitro and in vivo.^4^, ^6^ These findings indicate that inflammation is a potential therapeutic target to protect against obesity‐induced heart damage.

Pyroptosis refers to an inflammatory form of cell death, which is initiated by specific inflammasomes,^6^ such as canonical nucleotide‐binding oligomerization domain‐like receptor protein 3 (NLRP3) inflammasome, mainly consisting of sensor protein (NLRP3), adaptor protein (apoptosis‐associated speck‐like protein, ASC) and effector protein (procaspase‐1).^7^ Caspase‐1 is activated by NLRP3 inflammasome, and further cleaves gasdermin D (GSDMD) causing GSDMD‐NT oligomerization that forms large pores in the membrane, thereby leading to the release of mature IL‐1β and IL‐18.^8^ Recent studies have reported a potential association of pyroptosis with obesity‐related cardiac dysfunction and inflammation.^9^ For instance, palmitic acid (PA) was found to significantly increase the production of IL‐1β and IL‐18 in cardiac fibroblasts in vitro via activation of NLRP3 inflammasome.^10^ Additionally, NLRP3 inflammasome‐mediated pyroptosis was shown to be related to cardiac concentric remodeling in high‐fat diet (HFD)‐induced obese mice via modulation of systemic inflammation and metabolic disturbances.^11^ However, the precise mechanism by which obesity activates NLRP3 inflammasome‐mediated pyroptosis in cardiomyocytes is not well characterized.

Long noncoding RNAs (LncRNA), a class of noncoding RNA more than 200 nucleotides in length, are known to regulate gene expression at various levels (epigenetic regulation, transcriptional regulation, posttranscriptional regulation, etc.),^12^, ^13^ and are involved in almost all kinds of biological processes and pathways.^14^ Previous studies found a close relation between metastasis‐associated lung adenocarcinoma transcript 1 (MALAT1)^15^ and growth arrest‐specific transcript 5 (GAS5),^16^ two typical LncRNAs, with the PA‐induced myocardial inflammatory injury. Opa‐interacting protein 5‐antisense transcript 1 (OIP5‐AS1), an evolutionarily conserved LncRNA first discovered in zebrafish,^17^ plays important roles in various physiological processes, including carcinogenesis and cancer progression,^18^ myocardial ischemia reperfusion injury,^19^ diabetic nephropathy,^20^ and chronic obstructive pulmonary disease.^21^ However, whether OIP5‐AS1 plays a role in obesity‐induced myocardial injury and pyroptosis is still unknown.

Therefore, in this study, we investigated the impact of OIP5‐AS1 in obesity‐induced myocardial inflammation and pyroptosis. The results showed that downregulation of OIP5‐AS1 inhibited PA/HFD‐induced myocardial injury and pyroptosis. Mechanistically, it was found that OIP5‐AS1 acted as miR‐22 sponge to regulate NLRP3 inflammasome‐mediated pyroptosis.

## 2 MATERIALS AND METHODS

### 2.1 Cell cultivation and PA treatment

Rat myocardial cell line (H9c2) obtained from American Type Culture Collection (ATCC) was cultured with Dulbecco's modified eagle medium (DMEM) containing 10% fetal bovine serum at constant 5% CO_2_ level and 37°C. According to previous study,^22^ cells were treated with 400 μM PA for 24 h to establish obesity‐induced myocardial injury in vitro model.

### 2.2 Cell transfection

Small interfering RNA (siRNA) specifically targeting OIP5‐AS1 (si‐OIP5‐AS1), scrambled siRNA (negative control, NC), miR‐22 mimics, mimics NC, miR‐22 inhibitor, and inhibitor NC were obtained from Riobio Co. Ltd. They were transfected into cells using Lipofectamine 2000 (Invitrogen) according to the manufacturer's instructions. Twenty‐four hours or 48 h after transfection, cells were processed for next treatment and detection.

### 2.3 Oil red O staining

Cells were fixed with 10% formalin for 30 min, and then washed twice with phosphate‐buffered saline (PBS), followed by dipping in 60% isopropanol. Subsequently, cells were stained with Oil red O solution (Solarbio) for 10 min, washed twice with PBS, and observed under microscope.

### 2.4 Evaluation of membrane pores formation

Pyroptosis is characterized by formation of cell membrane pores. Thus, in the setting of pyropstosis, propidium iodide (PI) can penetrate through the membrane pores and stain the cell nucleus.^23^ Therefore, to observe membrane pores formation, cells were treated with PI (10 μM) for 30 min at room temperature, and then restained with 4′,6‐diamino‐2‐phenyl indole (DAPI). The percentage of PI‐positive cells was used to quantify cell pyroptosis.

### 2.5 Lactic dehydrogenase (LDH) release assay

Structural impairment of the cell membrane leads to release of LDH into the cell culture medium. Thus, LDH release is considered as a sensitive marker of cell pyroptosis.^24^ The level of LDH release was measured using LDH release assay Kit (Jiancheng Biological Engineering Research Institute, Nanjing, China) based on manufacturer's protocol.

### 2.6 Caspase‐1 activity assay

For this experiment, cells were digested by trypsase, centrifuged at 4°C, and collected after carefully sucking out the supernatant. The collected cells were mixed with lysis buffer, and then incubated in ice for 15 min. Following 16,000–20,000 g centrifugation at 4°C for 10–15 min, the supernatant was transferred to the centrifuge tube to detect caspase‐1 activity using Caspase 1 Activity Assay Kit (Beyotime).

### 2.7 IL‐1β and IL‐18 activity assay

The activity of IL‐1β and IL‐18 in culture medium or serum was detected using rat IL‐1β and IL‐18 enzyme linked immunosorbent assay (ELISA) Kit (Multi Science), based on the manufacturer's protocol.

### 2.8 RNA extraction and reverse transcription‐quantitative polymerase chain reaction (RT‐qPCR)

Total RNA was extracted using TRIzol reagent. Cytoplasmic and nuclear RNA was purified from the cytoplasmic and nuclear RNA fractions using Cytoplasmic and Nuclear RNA Purification Kit (Cat. 21000; Norgen). The isolated RNA was reverse‐transcribed into cDNA using a PrimeScript RT Reagent Kit (TaKaRa) and cDNA was subjected to real‐time qPCR amplification with the following specific primers:

OIP5‐AS1 forward: 5'‐GTGTTGTGGAGATTGAGGCAGGAG‐3'; OIP5‐AS1 reverse: 5'‐GGCAAGGTGAAGGACAGACAGC‐3'; GAPDH forward: (ssD1021‐1‐5, RIOBIO); GAPDH reverse: (ssD1022‐1‐5, RIOBIO).

qPCR was conducted by 7500 Real‐Time PCR Systems (ThermoFish) with SYBR Premix Ex Taq II (TaKaRa). Gene relative expression was analyzed using the $2^{-∆∆C_{t}}$ method.^25^

### 2.9 RNA pull‐down

Biotin‐labeled RNA probe was designed and synthesized by Riobio Co. Ltd. RNA pull‐down assay was performed as previously described^26^ using a Magnetic RNA‐Protein Pull‐Down kit (Pierce).

### 2.10 Luciferase assay

Wild‐type vector containing the binding region of OIP5‐AS1 with miR‐22 (pmirGLO‐OIP5‐AS1‐WT) and its match mutant vector (pmirGLO‐OIP5‐AS1‐Mut) were designed and synthesized by GenePharma Co. Ltd. miR‐22 mimics and the corresponding NC were cotransfected with pmirGLO‐OIP5‐AS1‐WT or pmirGLO‐OIP5‐AS1‐Mut, respectively. At 48 h posttransfection, luciferase activity was detected using a Dual‐Glo Luciferase Assay System (Promega).

### 2.11 Animal model and gene therapy

A total of 10 male Wistar rats (mean weight 120 ± 20 g) obtained from Beijing HFK Bioscience Co. Ltd. were fed with fat diet with 60 kcal% for 20 weeks to establish rat obesity model based on previous study.^27^ Adeno‐associated virus 9 (AAV9) vector containing short Hairpin RNA (shRNA) targeting OIP5‐AS1 (HBAAV9‐CTNT‐shOIP5‐AS1) and corresponding NC (HBAAV9‐CTNT‐sh‐NC) were constructed by Hanbio. Each rat was administered intravenous injection of 5 × 10^10^ particles AAV9 through the tail vein at 13th week of feeding period. To monitor the changes in cardiac function, rats were anesthetized with intravenous injection of pentobarbital sodium (30 mg/kg), and a rat Millar catheter was inserted into the left ventricle (LV) via the left common carotid artery to record the changes in heart rate, LV developed pressure (LVDP), and LV positive/negative first‐order derivative of ventricular pressure (±dp/dt) using a homodynamic system (MP150; BIOPAC Systems Inc.).

All animal experiments were performed in compliance with the Guide for the Care and Use of Laboratory Animals (NIH) and were approved by the Institutional Animal Care and Use Committee of the China Medical University (KT2018018).

### 2.12 Histological analysis

LV tissues were fixed with paraformaldehyde, dehydrated by passage through graded ethanol series, paraffin‐embedded, and cut into 5‐µm‐thick sections. The myocardial cross‐sectional area was measured using hematoxylin–eosin (HE) stained sections. Masson and Sirius red staining were employed to evaluate myocardial fibrosis and collagen deposition, respectively. The expressions of NLRP3 and cleaved caspase‐1 in the myocardium were detected using immunohistochemistry with primary antibodies against NLRP3 and cleaved caspase‐1 (NLRP3: 1:200, Proteintech Group; cleaved caspase‐1: 1:200, Proteintech).

### 2.13 Western blot analysis

Proteins were extracted from cells using RIPA lysis Buffer. The extracted proteins were quantified using the Enhanced BCA Protein Assay Kit (Beyotime), denatured by heat, isolated by SDS‐PAGE electrophoresis, and transferred onto PVDF membranes. After blocking with 1% bovine serum albumin solution for 1 h, the membranes were incubated overnight with primary antibodies (NLRP3: 1:1000, Proteintech; cleaved caspase‐1: 1:1000, Proteintech) at 4°C, followed by incubation with HRP‐conjugated IgG (1:5000; Proteintech). The membranes were exposed using FluorChem M Imaging systems. Relative densitometry was analyzed using Image J2x analysis software (NIH).

### 2.14 Statistical analysis

Data were presented as mean ± standard deviation (SD). Between‐group differences were assessed using the Student's *t* test. Multigroup comparisons were performed using one‐way analysis of variance followed by Fisher's least significant difference test. *p* Values < 0.05 were considered indicative of statistical significance. All statistical analyses were performed using SPSS version 17.0 software (SPSS Inc.).

## 3 RESULTS

### 3.1 PA activated pyroptosis and induced OIP5‐AS1 expression in cardiomyocytes

**Figure 1:**
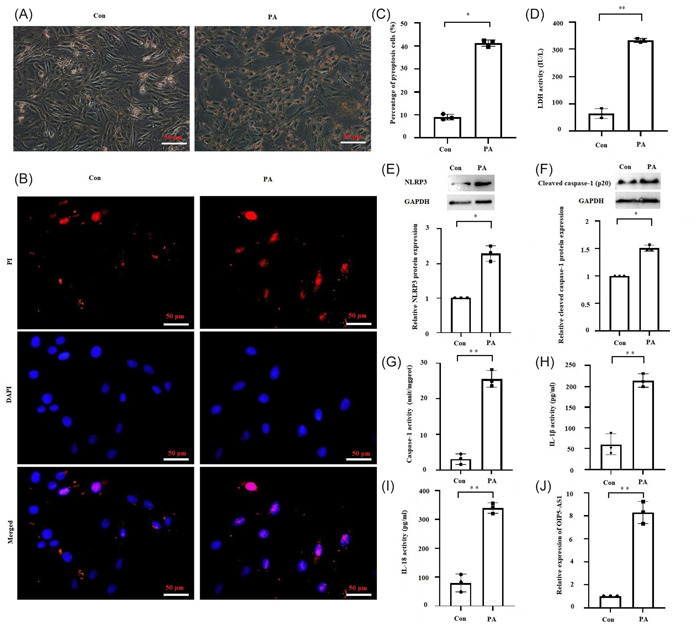
Palmitic acid (PA) activated pyroptosis and induced OIP5‐AS1 expression in cardiomyocytes. H9c2 cells were treated with 400 μM PA for 24 h. (A) Fat in H9c2 cells stained with oil red O solution. Scale bar = 50 μm. (B) and (C) Quantitative detection of propidium iodide (PI) staining reflecting the rate of cell pyroptosis. Scale bar = 50 μm. (D) Lactic dehydrogenase (LDH) released in cell culture medium. (E) and (F) Results of Western blot analysis showing protein expressions of NLRP3 and cleaved caspase‐1. (G) Measurement of caspase‐1 activity by colorimetry. (H) and (I) Results of enzyme linked immunosorbent assay (ELISA) showing IL‐1β and IL‐18 levels in culture medium. (J) Results of RT‐qPCR showing *OIP5‐AS1* expression in cardiomyocytes. Mean ± standard deviation values from three independent replicate experiments are presented. *p < .05; **p < .01.

Fat deposition was observed in H9c2 cells 24 h after 400 μM PA treatment (Figure 1A). Simultaneously, pyroptotic phenotype in PA‐treated H9c2 cells was also detected. PA‐treated cardiomyocytes showed membrane pore formation in H9c2 cells as evidenced by a significant increase in the rate of cells stained with PI and release of LDH (Figure 1B–D). The expression of NLRP3 and cleaved caspase‐1 protein, and the activity of caspase‐1, IL‐1β and IL‐18 were also remarkably increased after PA treatment (Figure 1E–I), suggesting that NLRP3 inflammasome‐mediated pyroptosis pathway was activated by PA. Correspondingly, the level of OIP5‐AS1 in the PA group was 8.29‐fold higher than that in the control group (Figure 1J). Collectively, these results suggested that saturated fatty acid activated pyroptosis and induced OIP5‐AS1 expression in cardiomyocytes.

### 3.2 Downregulation of OIP5‐AS1 inhibited PA‐induced pyroptosis in cardiomyocytes

**Figure 2:**
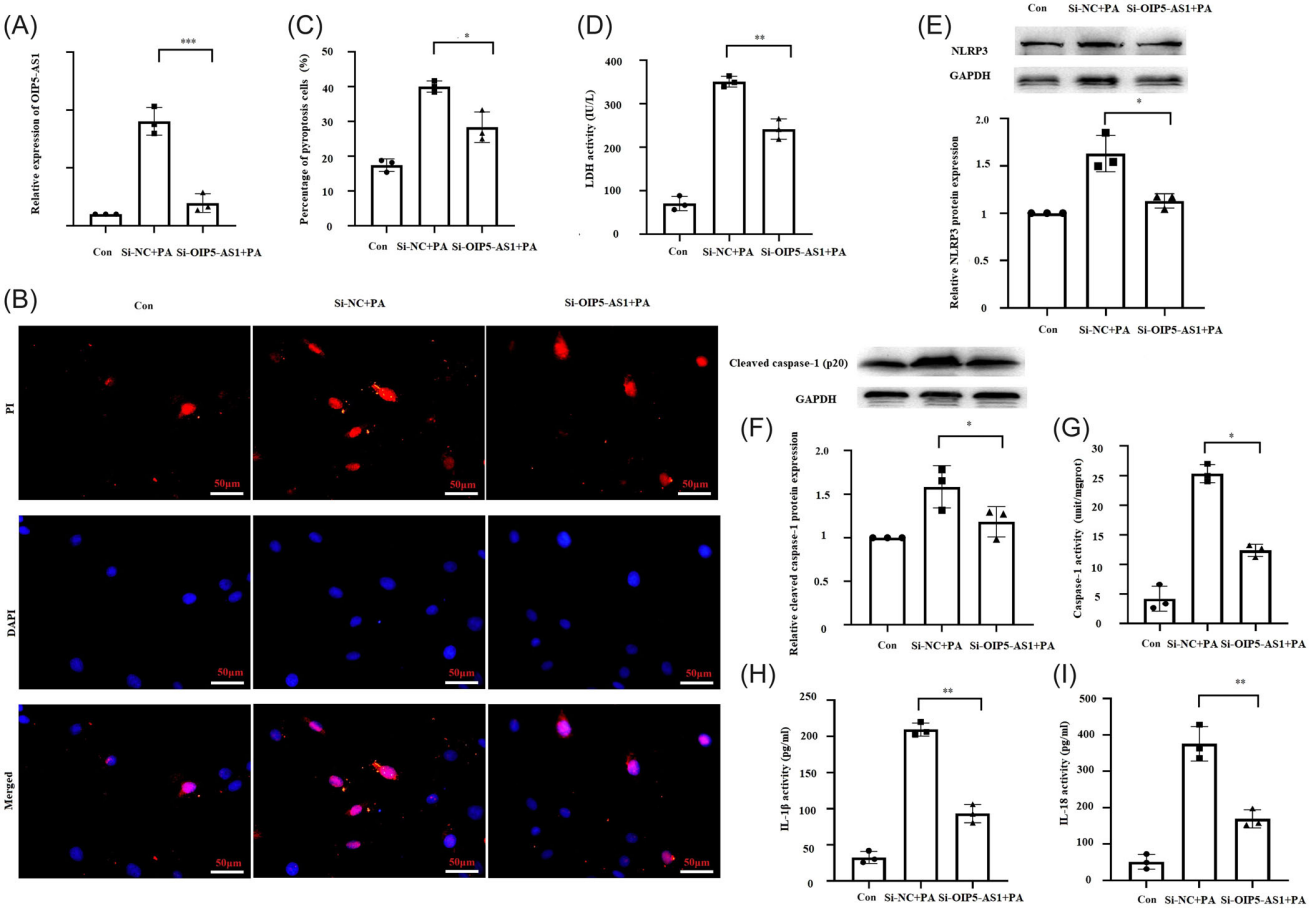
*OIP5‐AS1* knockdown inhibited palmitic acid (PA)‐induced pyroptosis in cardiomyocytes. H9c2 cells were transfected with small interfering RNA targeting OIP5‐AS1 (si‐OIP5‐AS1) and negative control (si‐NC) for 24 h, followed by treatment with 400 μM PA. Cells without any treatment were used as blank control (Con). (A) Results of RT‐qPCR showing *OIP5‐AS1* expression in cardiomyocytes. (B) and (C) Quantitative detection of propidium iodide (PI)‐stained cells indicating cell pyroptosis. Scale bar = 50 μm. (D) Lactic dehydrogenase (LDH) released in cell culture medium. (E) and (F) Results of Western blot analysis showing protein expressions of NLRP3 and cleaved caspase‐1. (G) Measurement of caspase‐1 activity by colorimetry. (H) and (I) Results of enzyme linked immunosorbent assay (ELISA) showing the levels of IL‐1β and IL‐18 in culture medium. Mean ± standard deviation values from three independent replicate experiments are presented. *p < .05; **p < .01; ***p < .001.

To examine the role of OIP5‐AS1 in saturated fatty acid‐induced pyroptosis, endogenous OIP5‐AS1 expression was notably repressed by transfection with si‐OIP5‐AS1 (Figure 2A). Furthermore, the percentage of cells affected by pyroptosis, release of LDH, the level of NLRP3 and cleaved caspase‐1 protein, and the activity of caspase‐1, IL‐1β, and IL‐18 were also significantly decreased by transfection with sh‐OIP5‐AS1 (Figure 2B–I). Collectively, these results suggested that downregulation of OIP5‐AS1 inhibited saturated fatty acid‐induced pyroptosis in cardiomyocytes.

### 3.3 OIP5‐AS1 bond to miR‐22

**Figure 3:**
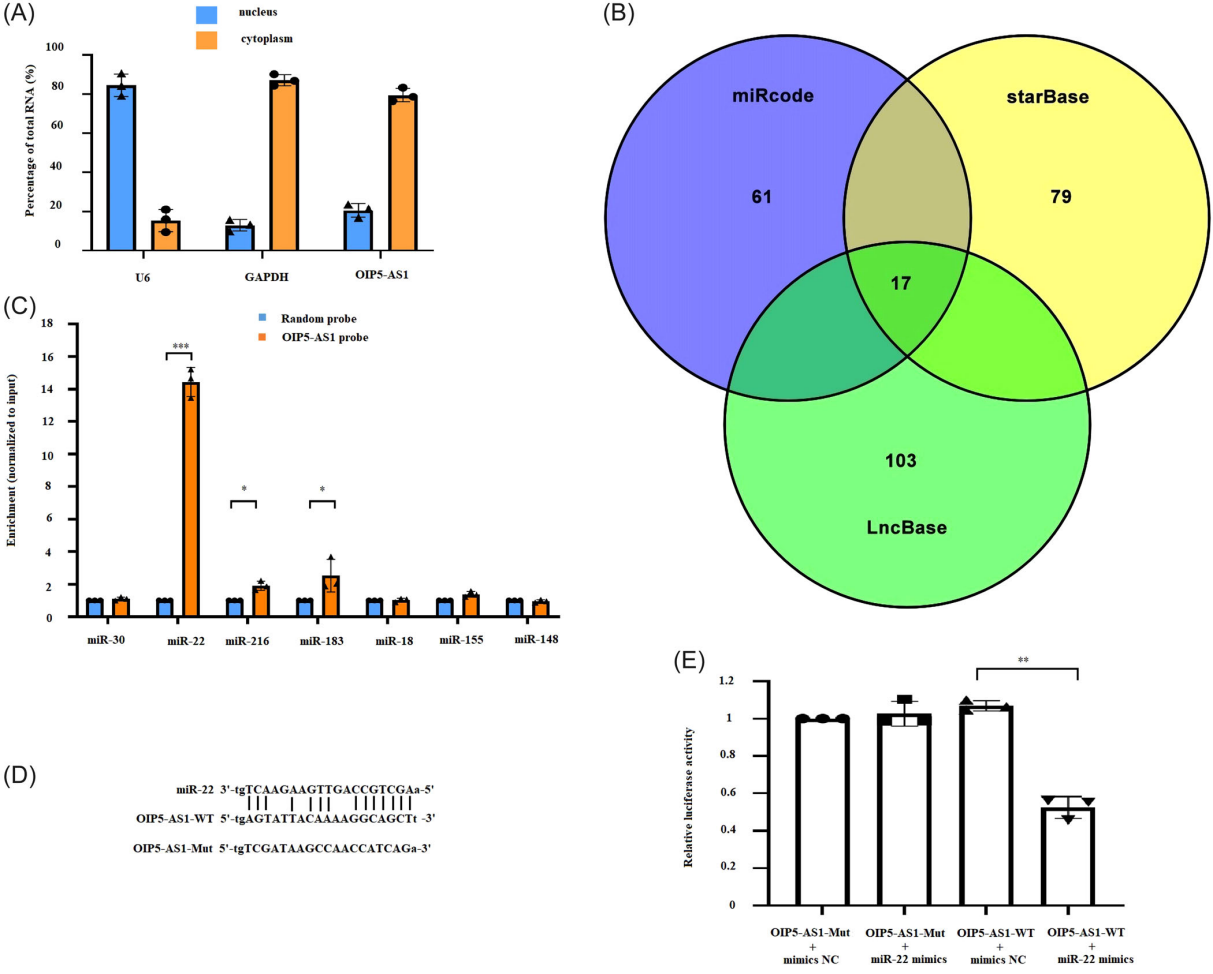
OIP5‐AS1 binds to miR‐22. (A) Localization of OIP5‐AS1 by nucleocytoplasmic separation assays. (B) miRNAs binding to OIP5‐AS1 were predicted using miRcode, starBase, and LncBase databases. (C) Potential binding miRNAs were pulled down using OIP5‐AS1 probe and quantitatively analyzed by RT‐qPCR. (D) and (E) Analysis of binding site between OIP5‐AS1 and miR‐22 using luciferase assay. Mean ± standard deviation values from three independent replicate experiments are presented. *p < .05; **p < .01; ***p < .001.

Considering that the mechanism for gene regulation by LncRNA is related to its cellular localization,^28^ the location of OIP5‐AS1 in cardiomyocytes was detected. The result of nucleocytoplasmic separation assays indicated that most of OIP5‐AS1 (nearly 90%) was located in the cytoplasm (Figure 3A). Therefore, microRNAs (miRNAs) binding to OIP5‐AS1 were predicted using miRcode, starBase, and LncBase databases. As shown in Figure 3B, 17 putative miRNAs binding to OIP5‐AS1 were predicted. Except for 10 miRNAs (miR‐29,^19^ miR‐26,^29^ miR‐223,^30^ miR‐218,^31^ miR‐153,^32^ miR‐141,^33^ miR‐140,^34^ miR‐137,^35^ miR‐129,^36^ and miR‐128^37^) that have already been reported to bind to OIP5‐AS1, the rest of 7 predicted miRNAs (miR‐30, miR‐22, miR‐216, miR‐183, miR‐18, miR‐155, and miR‐148) were detected in the pulled down product of OIP5‐AS1 probe. The results of RT‐qPCR indicated remarkable enrichment of miR‐22 expression (Figure 3C). Furthermore, the results of luciferase assay showed that cotransfection with miR‐22 mimics and wild‐type OIP5‐AS1 luciferase plasmid induced a significant decrease in luciferase activity. However, no significant difference was observed in luciferase activity by co‐transfecting miR‐22 mimics with mutated‐type OIP5‐AS1 luciferase plasmid (Figure 3D,E). Collectively, these findings demonstrated the specific binding of OIP5‐AS1 with miR‐22.

### 3.4 OIP5‐AS1 regulated NLRP3‐mediated pyroptosis via miR‐22

**Figure 4:**
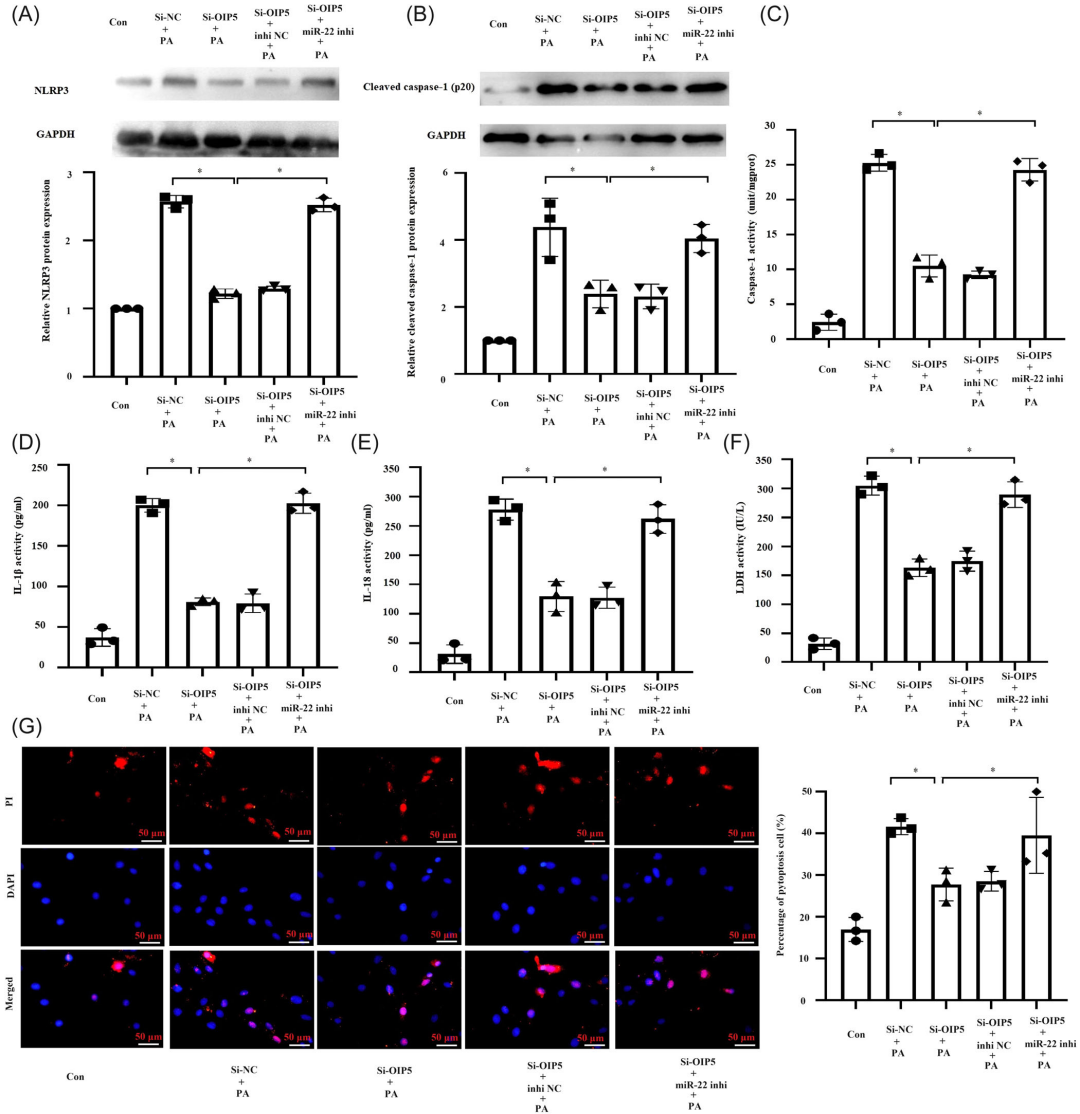
OIP5‐AS1 regulated NLRP3‐mediated pyroptosis via miR‐22. H9c2 cells were transfected with small interfering RNA targeting OIP5‐AS1 (si‐OIP5‐AS1) or negative control (si‐NC) together with miR‐22 inhibitor or inhibitor NC for 24 h, followed by treatment with 400 μM PA. Cells without any treatment were used as blank control (Con). (A) and (B) Results of Western blot analysis showing protein expressions of NLRP3 and cleaved caspase‐1. (C) Measurement of caspase‐1 activity by colorimetry. (D) and (E) Results of enzyme linked immunosorbent assay (ELISA) showing IL‐1β and IL‐18 levels in culture medium. (F) Lactic dehydrogenase (LDH) levels in cell culture medium. (G) Quantitative detection of propidium iodide (PI)‐stained cells indicating cell pyroptosis. Scale bar = 50 μm. *p < .05. Mean ± standard deviation values from three independent replicate experiments are presented. *p < .05.

Because *NLRP3* is a target gene of miR‐22,^38^ we investigated whether OIP5‐AS1 regulated NLRP3‐mediated pyroptosis via miR‐22. Treatment with miR‐22 inhibitor completely rescued the inhibition of NLRP3 by sh‐OIP5‐AS1 (Figure 4A). Moreover, suppression of cleaved caspase‐1 expression and caspase‐1 activity by sh‐OIP5‐AS1 were completely rescued by miR‐22 inhibitor (Figure 4B,C). Additionally, the repression of other pyroptotic phenotype by OIP5‐AS1 knockdown, including the release of IL‐1β, IL‐18, LDH, and the percentage of pyroptotic cells, were partially reversed by miR‐22 inhibitor (Figure 4D–G). These findings indicated that OIP5‐AS1 regulated NLRP3‐mediated pyroptosis via miR‐22.

### 3.5 Downregulation of OIP5‐AS1 ameliorated cardiac dysfunction in HFD rats

**Figure 5:**
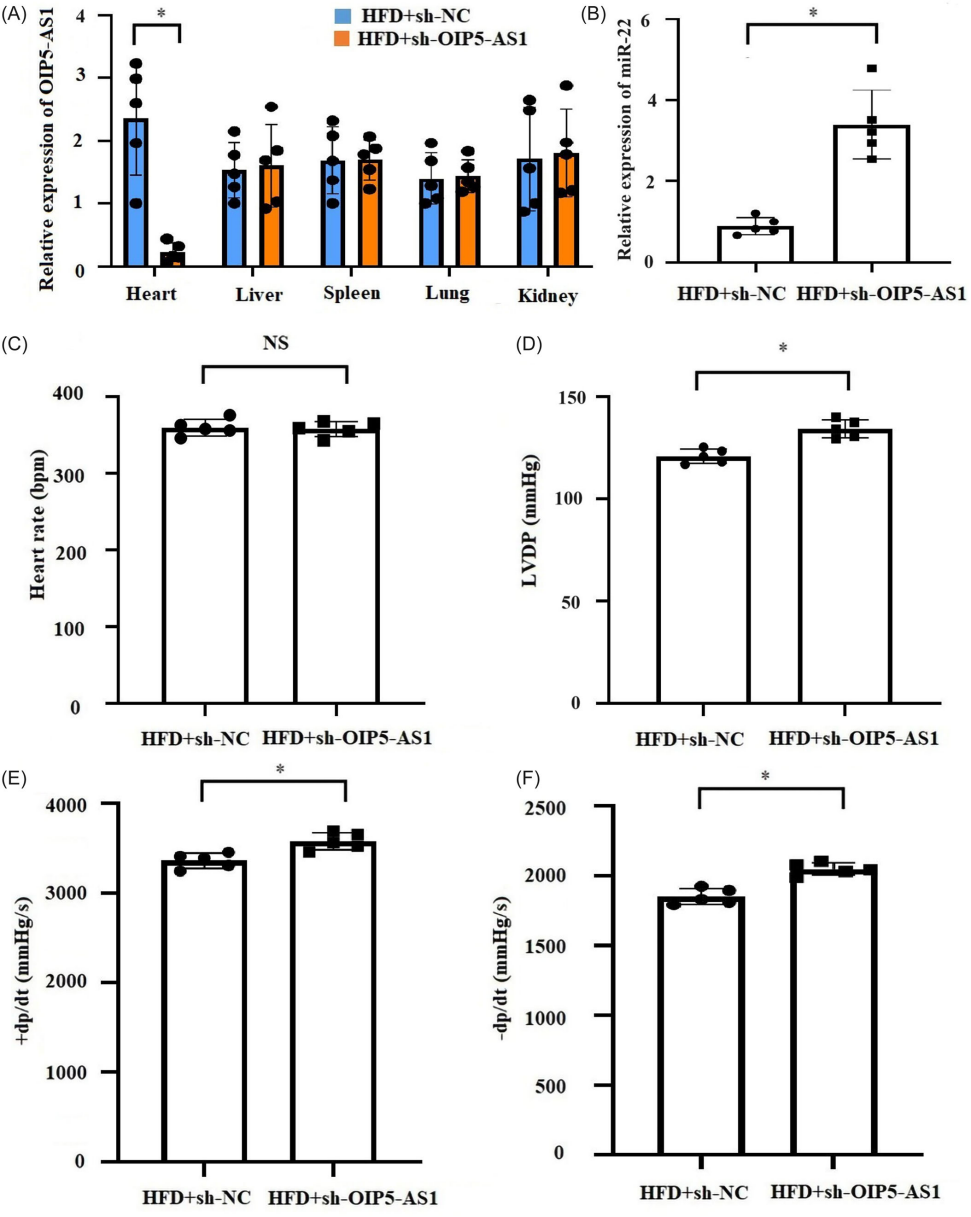
OIP5‐AS1 knockdown ameliorated HFD (high‐fat diet)‐induced cardiac dysfunction in vivo. OIP5‐AS1 was downregulated in the HFD‐fed rat heart through injecting with adeno‐associated virus 9 (AAV‐9)‐delivering short hairpin RNA targeting OIP5‐AS1 (sh‐OIP5‐AS1) or negative control (sh‐NC). (A) Relative expression of OIP5‐AS1 in heart, liver, spleen, lung and kidney was detected by RT‐qPCR, *n* = 5, *p < .05. (B) Relative expression of miR‐22 in heart was detected by RT‐qPCR, *n* = 5, *p < .05. (C–F) Cardiac functions, including heart rate, left ventricular developed pressure (LVDP), and positive/negative first‐order derivative of ventricular pressure (±dp/dt) were monitored using left ventricular incubation method. All data were expressed as mean ± standard deviation, *n* = 5, *p < .05.

To evaluate the impact of OIP5‐AS1 on obesity‐induced cardiac dysfunction in vivo, we examined the effect of inhibition of OIP5‐AS1 on cardiac function in HFD rats. As shown in Figure 5A, OIP5‐AS1 expression was significantly downregulated in heart by injection of HBAAV9‐CTNT‐shOIP5‐AS1, but no significant change in liver, spleen, lung, and kidney. Meanwhile, miR‐22 expression was remarkably upregulated in heart with injection of HBAAV9‐CTNT‐shOIP5‐AS1 (Figure 5B). Furthermore, OIP5‐AS1 repression led to a significant increase in the values of LVDP and ±dp/dt (Figure 5D–F), but there was no significant change in the value of HR (Figure 5C), which suggesting that downregulation of OIP5‐AS1 ameliorated HFD‐induced cardiac dysfunction.

### 3.6 Downregulation of OIP5‐AS1 ameliorated myocardial hypertrophy, fibrosis, remodeling, and pyroptosis in HFD rats

**Figure 6:**
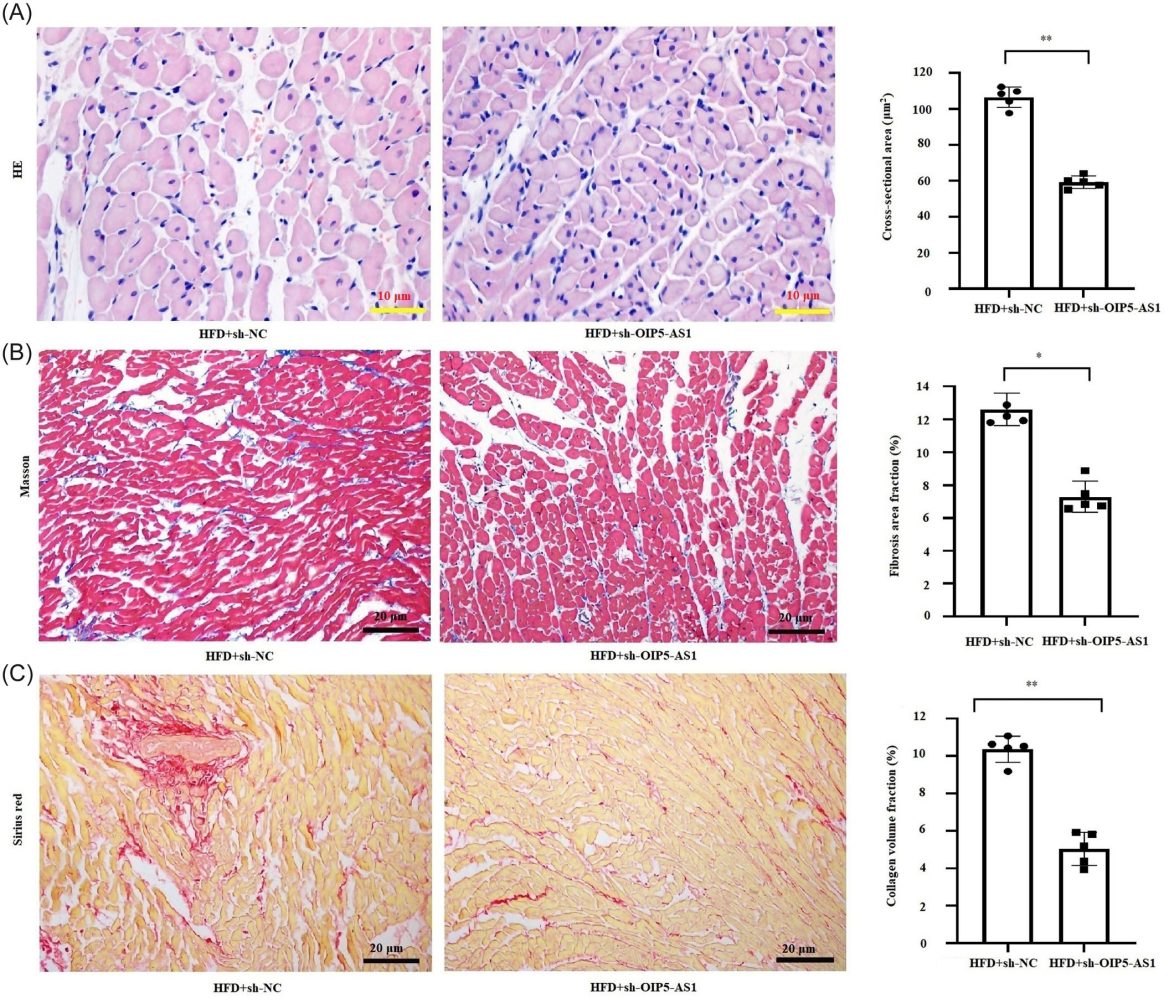
OIP5‐AS1 knockdown ameliorated HFD (high‐fat diet)‐induced myocardial hypertrophy, fibrosis and remodeling in vivo. OIP5‐AS1 was downregulated in the HFD‐fed rat heart through injecting with adeno‐associated virus 9 (AAV‐9)‐delivering short hairpin RNA targeting OIP5‐AS1 (sh‐OIP5‐AS1) or negative control (sh‐NC). (A) Measurement of myocardial cross‐sectional area by hematoxylin–eosin (HE) staining. Scale bar = 10 μm. (B) Evaluation of myocardial fibrosis by Masson staining. Scale bar = 20 μm. (C) Evaluation of myocardial collagen deposition by Sirius red staining. Scale bar = 20 μm. All data were expressed as mean ± standard deviation, *n* = 5, *p < .05.

**Figure 7:**
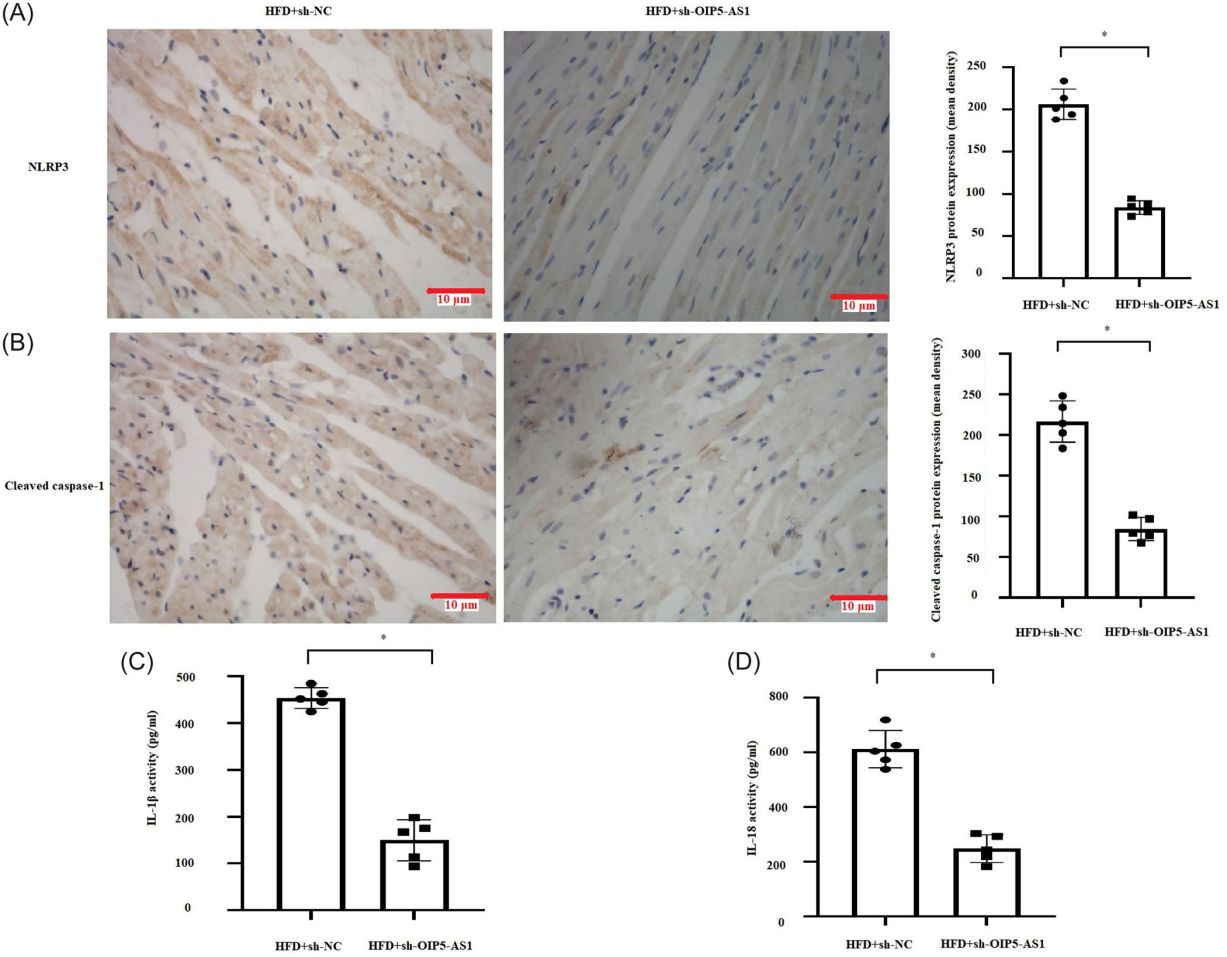
*OIP5‐AS1* knockdown suppressed HFD (high‐fat diet)‐induced pyroptosis in vivo. OIP5‐AS1 was downregulated in the HFD‐fed rat heart through injecting with adeno‐associated virus 9 (AAV‐9)‐delivering short hairpin RNA targeting OIP5‐AS1 (sh‐OIP5‐AS1) or negative control (sh‐NC). (A) and (B) Measurement of NLRP3 and cleaved caspase‐1 expression by immunohistochemistry. Scale bar = 10 μm. (C) and (D) Determination of serum levels of IL‐1β and IL‐18 by enzyme linked immunosorbent assay (ELISA). All data were expressed as mean ± standard deviation, *n* = 5, *p < .05.

To evaluate the impact of OIP5‐AS1 on obesity‐induced alteration of myocardial structure in vivo, we examined the effect of inhibition of OIP5‐AS1 on HFD‐induced myocardial hypertrophy, fibrosis, and remodeling. As shown in Figure 6, the cross‐sectional area, fibrosis area fraction, and collagen volume fraction in the myocardium of HFD‐rats were found to have significantly reduced by OIP5‐AS1 knockdown. In addition, the markers of pyroptosis including the expressions of NLRP3 and cleaved caspase‐1 in myocardium (Figure 7A,B) and the serum concentrations of IL‐1β and IL‐18 (Figure 7C,D) were notably decreased by OIP5‐AS1 knockdown in HFD rats. The above findings suggested that downregulation of OIP5‐AS1 ameliorated myocardial hypertrophy, fibrosis, remodeling, and pyroptosis in HFD rats.

## 4 DISCUSSION

OIP5‐AS1 has been shown to participate in multiple diseases and physiological processes.^18^, ^19^, ^20^, ^21^ However, its expression and function differed in various conditions. For example, *OIP5‐AS1* has been shown to be upregulated in the majority of cancers, and functions as an oncogene.^32^, ^36^, ^37^ In addition, studies have demonstrated high level of OIP5‐AS1 under condition of endothelial cell injury; moreover, OIP5‐AS1 knockdown showed a protective effect against endothelial injury.^39^, ^40^ On the contrary, OIP5‐AS1 is downregulated in myocardial infarct and diabetic cardiomyopathy, and overexpression of OIP5‐AS1 has been shown to alleviate ischemia/high glucose‐induced myocardial injury.^19^, ^41^ In the present study, PA was found to induce pyroptotic phenotype, and to simultaneously upregulate OIP5‐AS1 expression in cardiomyocytes, which reconfirmed that OIP5‐AS1 is a pyroptosis‐related lncRNA.^42^ Furthermore, OIP5‐AS1 knockdown inhibited PA/HFD‐induced pyroptosis, which is similar to the findings reported by Ji et al. wherein inhibition of OIP5‐AS1 repressed lipopolysaccharide (LPS)‐induced pyroptosis.^30^ Collectively, our finding suggests that OIP5‐AS1 is a novel therapeutic target for PA/HFD‐induced myocardial inflammatory injury.

There is now accumulating evidence that the action pattern of LncRNAs is closely related to their specific subcellular localization.^28^ In most cases, LncRNAs abundantly expressed in cytoplasm (also referred to as cytoplasmic lncRNA) have been found to interfere with the repression of miRNAs on its target gene as miRNAs sponges.^43^ In contrast, LncRNAs enriched in nucleus (nuclear lncRNAs) regulate chromatin organization or act as transcriptional regulators via binding to certain proteins, including chromatin modulating proteins, transcription factors, or RNA‐binding proteins (RBPs).^44^ Consistent with previous findings that OIP5‐AS1 is a cytoplasmic lncRNA,^45^ we also found that nearly 90% OIP5‐AS1 were distributed in cardiomyocytes cytoplasm. Thus, we investigated the interaction between OIP5‐AS1 and miRNA. In the present study, we demonstrated that miR‐22 can specifically bind to OIP5‐AS1, which is a novel finding of our study. Yang et al. found that overexpression of miR‐22 prevented hypoxia/reoxygenation (H/R)‐induced TNF‐α and IL‐6 in cardiomyocytes, and suggested that miR‐22 is a regulator for myocardial inflammatory response.^46^ Furthermore, miR‐22 was found to directly target NLRP3, and to regulate pyroptosis.^38^ More importantly, in several studies, HOTAIR^47^ and MALAT1,^48^ two well‐studied lncRNAs, were found to regulate NLRP3 inflammasome‐mediated pyroptosis via competitively binding miR‐22. Thus, we speculated whether OIP5‐AS1 regulated NLRP3‐mediated pyroptosis via miR‐22. As expected, our study demonstrated that OIP5‐AS1 regulated NLRP3 inflammasome‐mediated pyroptosis via competitively binding miR‐22, which may provide a new insight into the regulation of PA‐induced pyroptosis by OIP5‐AS1.

However, it should be emphasized that the suppression of IL‐1β, IL‐18, and LDH by OIP5‐AS1 knockdown was not thoroughly rescued by miR‐22 inhibitor, as compared with total reversion of NLRP3 and caspase‐1. This suggests the involvement of other pyroptotic signaling pathways in this process. For instance, recent studies have found that besides apoptosis, caspase‐3 can lead to the cleavage of GSDME to generate GSDME‐N fragment that pierce membranes, thereby causing pyroptosis.^49^ Thus, further studies are required to confirm whether OIP5‐AS1 affects other pyroptotic signaling pathways. In addition, we must acknowledge a limitation of our experiment that we did not employ NLRP3 inflammasome inhibitors or gene silencing techniques to confirm that myocardial damage is induced by activating the NLRP3 inflammasome pathway under PA stimulation in the mechanism research, which may limit the ability to directly attribute the observed myocardial damage to NLRP3 inflammasome activation.

**Figure 8:**
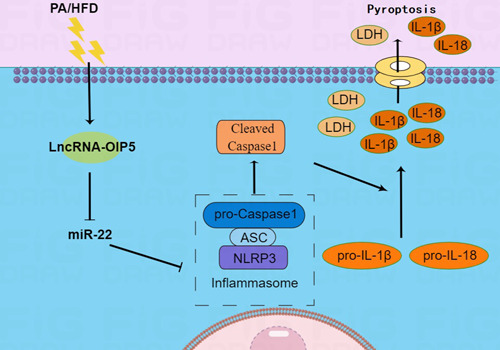
Palmitic acid (PA) and high‐fat diet (HFD) induce LncRNA‐OIP5‐AS1 expression and activates NLRP3 inflammasome‐mediated pyroptosis via miR‐22 in cardiomyocytes.

In summary, our study demonstrates that downregulation of OIP5‐AS1 can inhibit PA/HFD‐induced myocardial pyroptosis via the miR‐22/NLRP3 inflammasome axis (Figure 8). This finding may potentially lay a foundation for gene therapy for heart disease targeting *OIP5‐AS1*.

## AUTHOR CONTRIBUTIONS

**Qingxiong Yue**: Conceptualization; material preparation; experiments; data collection; data analysis; visualization; writing‐original draft. **Yan Liu**: Material preparation; experiments; data collection; data analysis; visualization; writing‐original draft. **Jun Ji**: Material preparation; experiments; data collection; data analysis. **Tao Hu**: Material preparation; experiments; data collection; data analysis. **Tong Lin**: Material preparation; experiments; data collection; data analysis. **Shuang Yu**: Material preparation; experiments; data collection; data analysis. **Shijun Li**: Conceptualization; experiments; data collection; data analysis; writing‐review and editing. **Nan Wu**: Conceptualization; experiments; data collection; data analysis; visualization; writing‐review and editing. All authors read and verified the underlying data in the study and approved the final manuscript.

## Data Availability

The data used to support the findings of this study are available from the corresponding author upon request.
